# The Distal Oblique Bundle in the Distal Forearm: From Anatomical Features to Clinical Implementation

**DOI:** 10.7759/cureus.50252

**Published:** 2023-12-10

**Authors:** Stavros Angelis, Emmanouil Apergis, Panagiotis Kanellos, Alexandros Apostolopoulos, Konstantinos Vlasis, Maria Piagkou, Dimitrios Filippou

**Affiliations:** 1 Anatomy, National and Kapodistrian University of Athens, Athens, GRC; 2 Orthopaedics, General Hospital Hellenic Red Cross Korgialenio - Benakio, Athens, GRC; 3 Upper Limb and Microsurgery, KAT General Hospital, Athens, GRC

**Keywords:** forearm, distal radioulnar joint, reconstruction, interosseous membrane, distal oblique bundle

## Abstract

Background and objective

The distal oblique bundle (DOB) is nowadays recognized as the thickest component of the distal interosseous membrane (DIOM). It is neither thought to be a clear-cut ligament, and nor does it follow the typical configuration of the rest of the DIOM. It is not always present and some studies have raised disputes about its prevalence and a few anatomical features. In this study, we aimed to provide data on the prevalence and anatomical features of the DOB, which are of great importance at this early stage of research into the topic. Our findings have been correlated with current knowledge and are expected to contribute to clinical implementation.

Materials and methods

Twenty-eight fresh-frozen forearms were utilized for measurements. Specifically, mean length, width, distance from the middle of the bundle’s insertion to the ulna to the tip of the styloid process of the ulna, as well as the distance from the midpoint of its insertion to the radius to the tip of the radiuses’ styloid process were calculated. The prevalence was described with a cutoff thickness point of 0.5 mm. Early results based on three cases of DOB reconstruction with the “Riggenbach” technique due to distal radioulnar joint (DRUJ) instability were documented.

Results

Eleven DOBs were reported out of the 28 specimens, suggesting a prevalence of 39.3%. The mean thickness was 0.88 mm (range: 0.6-1.3 mm), the mean width was 5.22 mm (range: 2.2-8.4 mm), and the mean length was 25.68 mm (range: 22.7-29.2 mm). Proximally, the mean distance from the bundle’s ulnar insertion to the tip of the styloid process of the ulna was 51.02 mm (range: 45.5-55.6 mm) while distally, the mean distance from the bundle’s insertion to the radius to the tip of the styloid process of the radius was 34.5 mm (range: 31.3-37.7 mm). After a follow-up of at least six months, improvement was evident in all measured areas in the three patients who underwent surgery. Additionally, they reported satisfaction and accomplishment of their preoperative goals.

Conclusions

Discrepancies in measurements in some anatomic features between studies are probably due to variations in specimen types, measurement methods, and sites. Efforts must continue to be made on a more extensive scale and in a more standardized manner for more factual results and conclusions. "Reconstruction-recreation" or "original construction-creation" procedures yield promising results in a fast, simple, and less invasive manner than traditional methods of DRUJ stabilization.

## Introduction

The distal oblique bundle (DOB) is currently considered the thickest component of the distal interosseous membrane (DIOM) [1,2]. It is not thought to be a clear-cut ligament; nor does it follow the configuration of the rest of the DIOM [2]. It has only recently been identified as a distinguishable structure; in 2009, Noda et al. recognized, named, and described its course, and associated it with the triangular fibrocartilage complex (TFCC) [1,3]. Their definition has been adopted in most of the relevant literature; many researchers agree on the DOB’s relationship with the TFCC, while Hohenberger et al. support the connection between the DOB and components of the TFCC [1,2,4-7]. As mentioned in a previous study by the same team of authors, we are convinced that it had already been detected by researchers in the past [1]. Probably, even though the structure had been described or even depicted by authors who had "stumped on" the DOB, they had never named it or realized its immense significance [1,6,8-13].

The stabilizing impact of the structure on the distal radioulnar joint (DRUJ) seems to be critical, especially when the TFCC function is rescinded for any reason [1,3,5,14]. Consequently, studies in the past 15 years have been vigorous and robust. Furthermore, the prevalence of the structure and the presence of certain anatomic features related to the DOB are the subject of reasonable disputes among researchers. This lack of consensus can pose challenges in clinical practice, especially since DOB is not always present and its absence can be instantly observed [1].

In this study, we sought to provide data on the prevalence and anatomical features of the DOB, which are of great importance at this early stage of research into the subject. We have correlated our research findings with current knowledge to facilitate and associate clinical implementation in three cases of DRUJ instability.

## Materials and methods

Our team of authors had conducted a study involving the analysis of 20 fresh-frozen forearms, obtained from eight male and two female Caucasians. These findings were combined with data from a previous study conducted by the same team in 2019, which included eight additional fresh-frozen forearms from three male and one female Caucasians [1]. Altogether, we used 28 upper limbs for our measurements, from 11 males and three females, 14 of which were left and 14 right. All sample measurements were conducted in the same manner and the same analogue vernier calliper. We also decided to include early results of three cases of DOB reconstruction with the “Riggenbach” technique to associate anatomical findings with clinical implementation [15,16].

**Figure 1:**
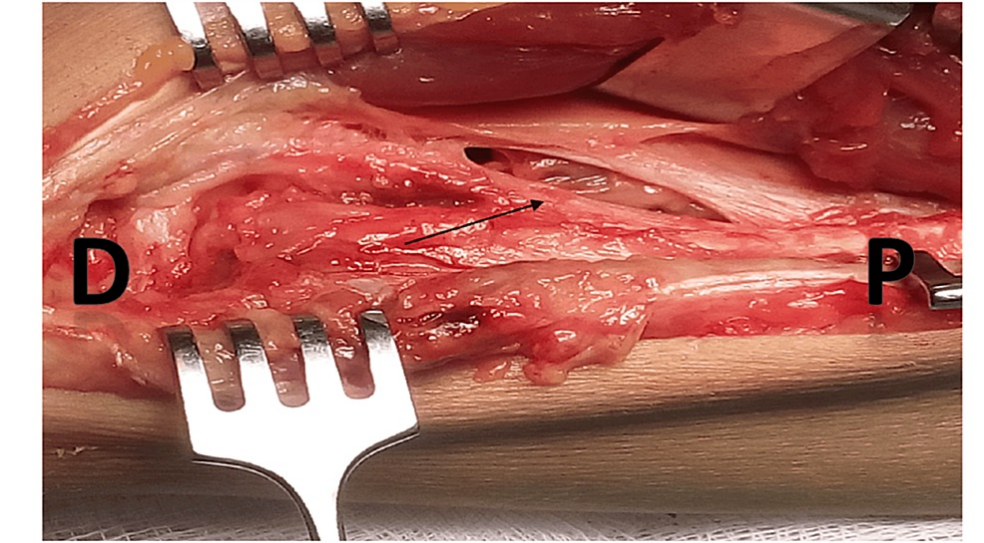
Distal oblique bundle (arrow) in the distal interosseous membrane of the forearm, originating proximally from the ulna and distally attaching to the radius D: distal forearm; P: proximal forearm

The mean age of the 28 fresh-frozen specimens (14 individuals) was 63.6 years (range: 45-77 years). Νo signs of pathology were observed on sites of interest. DIOM was cleared from all covering soft tissues and then investigated for the presence of a distinct DOB configuration. Structures were classified as DOBs if their thickness was 0.5 mm or greater [7,9,17]. Analogue vernier callipers (KS Tools Pocket Vernier Callipers 0-150 mm 300.0510) were used to carry out measurements with a precision of 0.05 mm. In the presence of a DOB, width and length would be assessed, while points of outgrowth and ingrowth to the ulna and radius respectively would be investigated (Figure 1). Particularly, the measurement of width was performed at the structure’s midpoint. Length was gauged from the midpoint of the bundle’s attachment to the ulna to the middle of its insertion to the radius. The distance between the middle of its attachment to the ulna to the tip of the styloid process of the ulna and the length between the midpoint of its attachment to the radius to the tip of the radiuses’ styloid process were measured.

**Figure 2:**
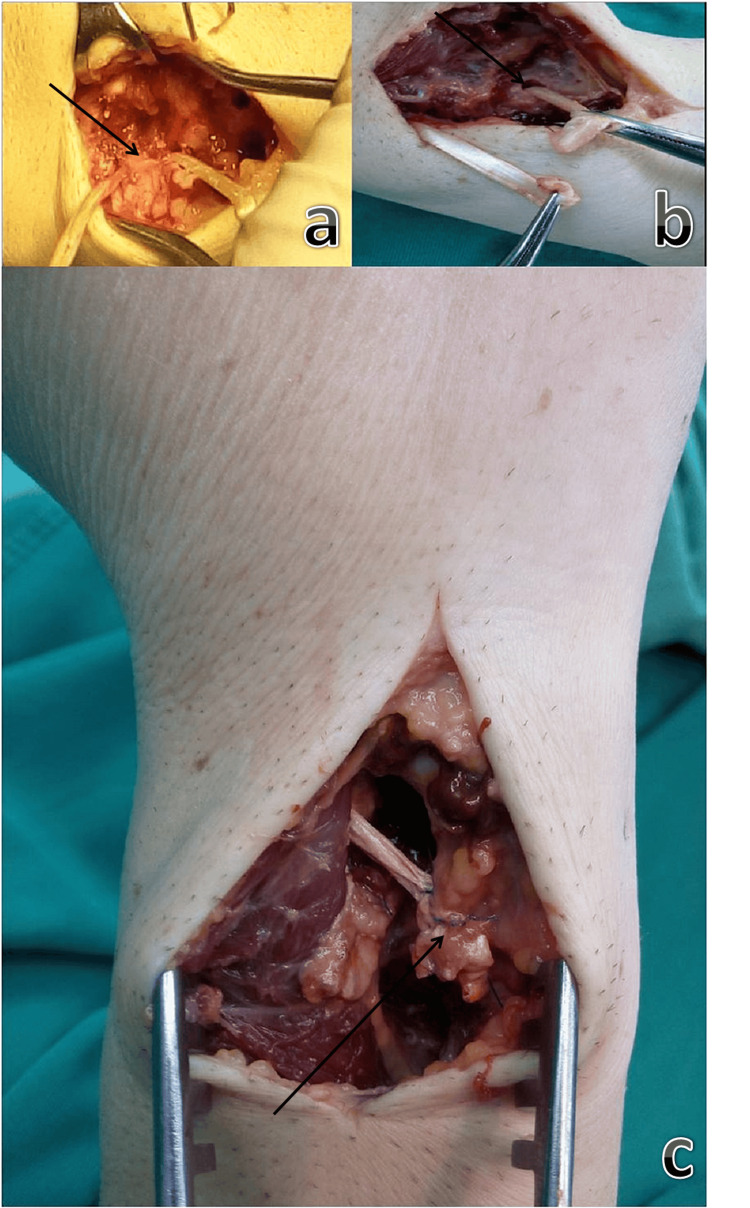
DOB reconstruction with the use of a palmaris longus tendon graft a. The radial hole is drilled just inferior to the sigmoid notch. A minimum bone bridge of 3-5 mm is intentionally preserved to ensure stability and integration of the graft with the host bone (arrow) b. The ulnar hole is oriented obliquely along the dorsal aspect of the ulna and directed volarly and radially (arrow). The graft is threaded through the holes where the DOB, if present, is naturally anchored c. A Pulvertaft weave is created between the two ends of the graft (arrow) DOB: distal oblique bundle

Regarding the three cases of DOB reconstruction, employing the Riggenbach et al. technique, we performed a dorsal approach through the fifth compartment of the wrist to reconstruct the bundle by using a palmaris longus tendon graft. The graft was passed through holes created with a 3.0 mm drill at the ulna and radius at the site of the dorsal oblique bundle (Figure 2). Specifically, the ulnar aperture was situated on the top of the ulna, angled diagonally and pointing towards the palm and the thumb side to emerge at the interosseous membrane level. A radial aperture was drilled slightly proximally to the sigmoid notch and along the dorsal aspect of the radius. A minimum bone bridge of 3-5 mm, both in the radius and the ulna, was intentionally preserved to ensure stability and integration of the graft with the host bone [15,16]. The graft was threaded through the holes and a Pulvertaft weave was created between the two ends. The suturing was performed with the hand in 60^o^ of supination while tension was set during the first pass [15,16].

This technique was employed in three cases of DRUJ instability resulting from distal radius fractures healed conservatively after closed reduction. Following the surgery, a brachiopalmar splint was utilized to immobilize the arm in supination for four weeks, followed by the use of an antebrachial splint for another four weeks. After the cast was removed, range of movement exercises were permitted, and strengthening exercises were initiated after 12 weeks.

The patients comprised two females and one male with a mean age of 59.6 years. All patients had suffered a fracture of the distal radius in their right dominant hand and were followed up for at least one year before surgical reconstruction of the DOB was chosen. In all patients, the ipsilateral palmaris longus graft was employed. Preoperative mean quick disability of the arm, shoulder, and hand (QuickDASH) score was 65 while the mean grip strength was 17.6 kg in their injured right dominant hand. The mean grip strength of their left non-dominant uninjured hand was 46.3 kg. The mean joint balance of the wrist was 58.3° in flexion and 75° in extension, while the forearm's mean supination capacity was 60° and the mean pronation was 73.3°.

## Results

Among the 28 freshly frozen forearms examined, we identified 11 DOBs, revealing a prevalence rate of 39.3%. Six were found on male right hands and three on left hands. Two males had DOBs on both hands. One DOB was observed on a female right hand and one on another female left hand. The mean age of the 11 fresh-frozen specimens with a DOB was 60.9 years (range: 45-74 years). The measured mean thickness was 0.88 mm (range: 0.6-1.3 mm). Similarly, the average width was 5.22 mm (range: 2.2-8.4 mm), while the mean length was 25.68 mm (range: 22.7-29.2 mm. The bundle's ulnar insertion had a mean distance of 51.02 mm (range: 45.5-55.6 mm) from the tip of the styloid process of the ulna, while the middle of the bundle's insertion to the radius had a mean distance of 34.5 mm (range: 31.3-37.7 mm) from the tip of the styloid process of the radius. For detailed measurements, please refer to Table 1.

**Table 1:** Summary of measurements *The last row represents the mean values of the studied variables M: male; F: female; R: right; L: left

	Sex	Hand	Age, years	Thickness	Width	Length	Ulnar insertion	Radial insertion
1	M	R	65	0.9	4.3	25.6	53.4	37.7
2	M	R	45	1.0	6.2	27.5	51.9	31.3
3	F	L	52	0.6	2.2	23.2	45.5	33.1
4	F	R	73	0.8	3.4	23.8	50.1	32.4
5	M	R	63	0.6	4.0	22.7	46.4	34.0
6	M	R	74	0.8	5.8	24.1	46.7	36.1
7	M	R	62	1.3	8.4	26.3	53.4	35.5
8	M	L	62	1.1	7.5	26.1	53.3	35.7
9	M	R	55	0.9	6.1	28.3	55.6	34.8
10	M	L	55	1.0	5.7	29.2	53.2	34.6
11	M	L	64	0.7	3.8	25.7	51.7	34.4
Mean values*	-	-	60.9	0.88	5.22	25.68	51.02	34.50

Regarding the "Riggenbach et al." reconstruction, the three patients had been monitored for at least six months (between six and 13 months) [15,16]. Measurements at six months postoperatively were as follows: the mean Quick-DASH score was 16.1 while the mean grip strength was 33.3 kg in their injured right dominant hand. The mean joint balance of the wrist was 78.3° in flexion and 85° in extension, while the forearm's mean supination capacity was 73.3° and mean pronation was 83.3°. All patients reported improvement as far as pain and function were concerned and provided informed consent for the publication of these results.

## Discussion

The DOB is nowadays widely accepted among experts to be a secondary stabilizer of the DRUJ [1-3,5,14]. Noteworthy attempts with very promising results have been employed to "reconstruct-recreate" or even "originally construct-create" the configuration in cases of instability. This requires a thorough familiarity with the anatomy of the DOB and DRUJ. Therefore, research on the prevalence and anatomical traits is vital for accurate representation and sounder results. Most authors seem to agree on the definition and ties to the TFCC as described by Noda et al. [3]. However, data so far seem to suggest some disparity that probably has to do with the variety in volumes and types of specimens, and different means, sites, and practices of measurement.

The prevalence of the DOB on the forearm demonstrates a noticeable discrepancy [1,2]. In the early stages, when it was first established as a distinct anatomical structure, the DOB was thought to be encountered in 40% of the general population [3,14,17]. Our results seem to agree; we have found 11 DOBs on 28 forearms and documented a prevalence of 39.3%. Dy et al. and Delbast et al. have reported a prevalence of 50% in 10 and 12 fresh frozen specimens respectively [18,19]. However, all the aforementioned research was based on small samples (10-30 forearms). Admittedly, Hohenberger et al. seem to have dissected a representative volume of specimens (185 forearms). They reported 53 DOBs and a prevalence of 29% [7]. However, they utilized cadaveric hands rather than freshly frozen ones. According to the authors, the process of embalming may have caused a reduction in volume, potentially impacting thickness measurements [1,2,7]. On the other hand, Bin Abd Razak et al. reported an incidence of 44% in a study of 25 cadaveric forearms. However, no detail about the standards of measurement of the DOB was shared [20]. In their study, Kim et al. analyzed MRI measurements of 80 specimens and reported a prevalence of 32.5% [21].

To our knowledge, this is the only study reporting prevalence similar to the one documented by Hohenberger et al. Nevertheless, only structures at least 1.0 mm thick are considered DOBs in this paper. This suggests that should the reference point be 0.5 mm, prevalence results would likely be much higher. Similarly, Lee et al. and He et al. reported an incidence of DOBs of 34% and 20.7% in large samples of 85 and 121 living forearms with the use of MRI and structures thicker than 1.0 mm respectively [22,23]. Surprisingly, studies have lately tended to report a much higher incidence of the DOB. For example, Low et al., Rein et al., and Kholinne et al. found the structure in seven out of 10 (70%), 10 out of 11 (91%), and 14 out of 16 (87,5%) freshly frozen forearms respectively [24-26].

Besides the type of specimen and mean of measurement, the prevalence may partly have to do with the age of the samples. As known, during aging, the loss of volume in musculoskeletal structures is rapid; therefore, we can presume that the presence of DOB (thickness greater than 0.5 mm) in younger samples will be higher. Dy et al.'s study, where the average age of the samples is only 51 years, reports an incidence rate of 50%, while Noda et al. and Kitamura et al. reported an incidence of 40%, but the average age of the samples was clearly higher (85 and 79 years respectively) [3,17,18]. The mean age of our samples was 60.9 years, which probably does not support this theory. A third possible reason for the difference in measurements between samples could be the inhomogeneity between the right and left hands [2]. Specifically, Hohenberger et al. discovered the structure in 41 right and 12 left forearms [7]. Hence, there is probably a predominance of the structure in the right upper extremity. Of course, in their sample, right hands were somewhat dominant, but a rational question that arises is whether DOB is indeed dominant in the right upper extremities compared to the left. Considering that most of the population is right-handed, one could link the prevalence of DOB in the right upper extremities to the more frequent use of the right hand than the left.

Finally, we should probably examine possible differences in the frequency of the presence of DOB that may exist between men and women. Among the studies mentioned, none emphasizes this difference except the one by Dy et al. where DOBs were observed in 25% (1/4) of female samples and 66% (4/6) of male samples [18]. Of course, due to the small sample, conclusions are precarious and could easily be attributed to chance. On the other hand, Kim et al. reported no statistically significant difference in the presence of DOB between men and women [21]. Nonetheless, it is our theory that if the presence and development of DOBs are related to manual work, a difference between men and women will probably arise. Assuming that most of the population is right-handed and manual labor is usually carried out by males, presumably the development of the DOB is related to the exercise of the upper limb [2]. We reported two DOBs in female and nine in male samples, while seven were found on right hands and four in left ones.

As far as anatomical features are concerned, the authors tend to agree on most. Specifically, length (24-26 mm), ulnar outgrowth (approximately 50 mm from the tip of the ulnar styloid), and ingrowth to the radius (approximately 35 mm from the tip of the radial styloid process) are more or less consistent [2]. Our measurements are also in line with this; we report a mean length of 25.68 mm (range: 22.7 to 29.2 mm). Only Rein et al. reported a mean length of 17.5 mm but underlined possible differences in measurement practices [25]. Proximally, the mean distance from the middle of the bundle’s ulnar insertion to the tip of the styloid process of the ulna was 51.02 mm (range: 45.5-55.6 mm), while distally, the mean distance from the middle of the bundle’s insertion to the radius to the tip of the styloid process of the radius was 34.5 mm (range: 31.3-37.7 mm). On the other hand, there seems to be a discrepancy concerning thickness [1,2]. The largest sample registered reported a mean thickness of 0.9 mm (range: 0.5-1.8 mm) [7]. We present similar results (0.88 mm, range: 0.6-1.3 mm) and so do Dy et al. (0.85 mm ± 0.28 mm, range: 0.64-1.33 mm) [18]. However, Noda et al. (1.5 mm), Moritomo (1.2 mm), Kitamura et al. (1.2 mm), Rein et al. (1.2 mm), and Okada et al., in their ultrasound research of 14 forearms (1.09 mm), seem to disagree [3,9,17,25,27]. Kim et al., who employed 1.0 mm as their reference point, documented a mean thickness of 1.4 mm (range: 1.1-1.7 mm) in the DOB group and 0.6 mm (range: 0.2-0.9 mm) in the non-DOB group [1,21].

Finally, mean width measurements often demonstrate a wide disparity. As Kitamura et al. noted, the bundle is not always linear and thick, but may sometimes offer some variations in its morphology [1,17]. We have experienced some of those divergencies in our sample and noticed a high range between widths. After performing our width measurements in the middle of the structure, we report a mean width of 5.22 mm (range: 2.2-8.4 mm), which approximately aligns with the findings by Noda et al. (4.4 mm) and Kitamura et al. (5,1 mm), whereas Noda et al. (9.0 mm) and Rein et al. (9.4 mm) reported a significant difference in their mean width measurements [25].

As mentioned, the interest in DOB has tended to grow due to practical and minimally invasive open or percutaneous techniques that have been implemented for the past decade [15,16,28,29]. Once the structure's usefulness as a secondary isometric stabilizer of the DRUJ was established, it was a natural consequence to invent means to mimic its function. The principles of "reconstruction-recreation" or even "original construct-creation" are simple, and procedures offer a low learning curve and are less time-consuming and less invasive than traditional DRUJ stabilization techniques [2]. More importantly, the results seem more than promising. Our small experience with three cases of DOB "re-construction" so far has been very encouraging. Obvious improvement has been observed in all objective and calculable areas, and patients also report satisfaction and achievement of preoperative goals.

Limitations

It is important to note that this study has certain inherent limitations. The primary limitation is the relatively small sample size. In particular, the sample of three DOB reconstructions is probably trivial. However, the comparison of results with those of other researchers and the application of anatomical knowledge to clinical cases have enabled us to draw valuable conclusions. The study involved a total of 28 upper limbs collected from 14 donors. Due to the small sample size, statistical power could not be determined before the beginning of the study. Furthermore, the evaluation of both limbs minimized the variation in the results obtained from the anatomical analysis. Although the thickness of the DOB is commonly used to assess its role in joint stability, other morphological features such as width and insertion location may also be significant. For consistency with the literature, we utilized the thickness criterion to identify a unique DOB.

Another limitation pertains to the fact that pre- and postoperative evaluations of DRUJ stability on the three patients were conducted solely through physical examination. No additional diagnostic tests or imaging were used to assess joint stability. Another limitation of the study is that despite randomizing the reconstructions, the investigators were not blinded to the study protocol during clinical testing. However, objective measures were used to standardize the clinical testing process. It is worth noting that this particular study did not take into account other anatomical structures, such as the pronator quadratus or extensor carpi ulnaris, which could have played a role in maintaining the stability of the DRUJ. It is important to clarify that our study, being cross-sectional, cannot establish a causal relationship between the DOB and DRUJ instability, and this was not the purpose of our research. Nevertheless, clinically positive results after "reconstruction-construction" indicate its consequential role in stabilizing the DRUJ.

## Conclusions

The DOB is the thickest part of the DIOM of the forearm; when present, it acts as a secondary isometric stabilizer. Most of the authors seem to agree on measurements of most anatomic features except thickness and width. The prevalence is also an issue of debate. Partly, this is due to variations in specimen types, measurement methods, and sites. Efforts must continue to be made on a more extensive scale and in a more standardized manner to obtain more factual results and conclusions. "Reconstruction-recreation" or "original construction-creation" procedures yield promising results in a fast, simple, and less invasive manner.
